# Scalable, semi-automated fluorescence reduction neutralization assay for qualitative assessment of Ebola virus-neutralizing antibodies in human clinical samples

**DOI:** 10.1371/journal.pone.0221407

**Published:** 2019-08-27

**Authors:** Elena N. Postnikova, James Pettitt, Collin J. Van Ryn, Michael R. Holbrook, Laura Bollinger, Shuǐqìng Yú, Yíngyún Caì, Janie Liang, Michael C. Sneller, Peter B. Jahrling, Lisa E. Hensley, Jens H. Kuhn, Mosoka P. Fallah, Richard S. Bennett, Cavan Reilly

**Affiliations:** 1 Integrated Research Facility at Fort Detrick, National Institute of Allergy and Infectious Diseases, National Institutes of Health, Research Plaza, Frederick, MD, United States of America; 2 Coordinating Centers for Biometric Research, Division of Biostatistics, University of Minnesota, Minneapolis, MN, United States of America; 3 Laboratory of Immunoregulation, National Institute of Allergy and Infectious Diseases, National Institutes of Health, 10 Center Dr, Bethesda, MD, United States of America; 4 Emerging Viral Pathogens Section, Laboratory of Immunoregulation, Division of Intramural Research, National Institute of Allergy and Infectious Diseases, National Institutes of Health, Research Plaza, Fort Detrick, Frederick, Maryland, United States of America; 5 National Public Health Institute of Liberia, Monrovia, Liberia; Tulane University, UNITED STATES

## Abstract

Antibody titers against a viral pathogen are typically measured using an antigen binding assay, such as an enzyme-linked immunosorbent assay (ELISA), which only measures the ability of antibodies to identify a viral antigen of interest. Neutralization assays measure the presence of virus-neutralizing antibodies in a sample. Traditional neutralization assays, such as the plaque reduction neutralization test (PRNT), are often difficult to use on a large scale due to being both labor and resource intensive. Here we describe an Ebola virus fluorescence reduction neutralization assay (FRNA), which tests for neutralizing antibodies, that requires only a small volume of sample in a 96-well format and is easy to automate. The readout of the FRNA is the percentage of Ebola virus-infected cells measured with an optical reader or overall chemiluminescence that can be generated by multiple reading platforms. Using blinded human clinical samples (EVD survivors or contacts) obtained in Liberia during the 2013–2016 Ebola virus disease outbreak, we demonstrate there was a high degree of agreement between the FRNA-measured antibody titers and the Filovirus Animal Non-clinical Group (FANG) ELISA titers with the FRNA providing information on the neutralizing capabilities of the antibodies.

## Introduction

Ebola virus (EBOV) is a single-stranded negative-sense RNA virus of the mononegaviral family *Filoviridae* [1]. Since its discovery in 1976, EBOV has caused at least 18 outbreaks of human Ebola virus disease (EVD) in Equatorial Africa with an overall average case-fatality rate of ~41% [2–4]. The largest recorded EVD outbreak occurred from 2013 to 2016 in Western Africa and encompassed 28,652 human infections and 11,325 deaths [5]. This outbreak provided an opportunity to study EVD patients, survivors, and their contacts. Presence of anti-EBOV antibodies in blood is presumed to be a good measure for previous or ongoing infection to EBOV or treatment with medical countermeasures such as vaccines [6–8]. Hence, methods to reliably, sensitively, and rapidly detect specific anti-EBOV neutralizing antibodies from pre-clinical and clinical trials are needed.

Two enzyme-linked immunosorbent assays have been deployed to detect anti-EBOV antibodies (an immune correlate of protection) in EVD survivors and vaccinees—the commercially available Alpha Diagnostics International ADI (ELISA) and the Filovirus Animal Non-clinical Group (FANG) ELISA [9]. Both assays detect anti-EBOV IgG targeting a single viral protein, but do not provide information on the functionality or the ability to neutralize virus, of the antibodies in each sample. In addition, as the antibody response shifts from IgM to IgG production, researchers may miss important early immune correlates by only measuring one antibody isotype against a single protein.

Assays which measure live virus neutralization provide insight into the functionality of the total humoral immune response generated following either natural EBOV infection or vaccination. The plaque-reduction neutralization test (PRNT) is a reliable technique for determination of neutralizing antibody titers of viruses in general, but PRNT for EBOV is labor-intensive, difficult to scale, and may take 7–8 days to complete [7, 8, 10, 11]. Here we present a semi-automated fluorescence reduction neutralization assay (FRNA) as an easier and faster alternative to PRNT and use FRNA to detect EBOV-neutralizing antibodies in animal samples or human clinical samples.

## Materials and methods

### Cells and virus

Ebola virus/H.sapiens-tc/GIN/2014/Makona-C05 (EBOV) was kindly provided by Dr. Gary P. Kobinger (National Microbiology Laboratory, Canadian Science Centre for Human and Animal Health, Public Health Agency of Canada, Winnipeg, Manitoba, Canada) [12]. The virus was passaged at Integrated Research Facility-Frederick two times in grivet (*Chlorocebus aethiops*) kidney epithelial Vero E6 cells (BEI Resources, # NR596, Manassas, VA, USA).

### Fluorescence reduction neutralization assay

The FRNA was initially established using a commercially available EBOV antiserum. Briefly, polyclonal rabbit anti-EBOV-like particle antiserum (IBT Bioservices #01–0004) was serially diluted (1:40–1:20480) in serum-free Dulbecco’s modified Eagle’s medium (DMEM, Lonza # 11965118) to represent a serially diluted clinical sample. The surrogate sample dilutions were heat-inactivated at 56°C for 1 h prior to use. EBOV was diluted in serum-free DMEM and mixed 1:1 (vol/vol) with each surrogate. The virus/sample mixtures were incubated at 37°C in a humidified 5% CO_2_ atmosphere for 1 h to allow anti-EBOV antibodies to bind virions. The virus/sample mixtures were then transferred to wells of a 96-well plate (Greiner Bio-One #655948) containing Vero E6 cells at 90–100% confluency (seeded at 40,000 cells/well), resulting in a final target titer of 50,000 plaque-forming units/well (multiplicity of infection, MOI = 1.25), and incubated at 37°C and 5% CO_2_ for 48 h.

After 48 hours of incubation, cells were fixed by adding 2x neutral-buffered formalin (Thermo Scientific # 23-751-800, Kalamazoo, MI) directly to the media for 30 min at room temperature. Plates were stored in 10% neutral-buffered formalin overnight at 4°C to inactivate virus prior to staining. Cells were washed twice with phosphate-buffered saline (PBS) diluted in water purified through reverse osmosis to 1X (Fisher Scientific, Phosphate Buffered Saline, PBS, 10X, # BP3994) and then permeabilized with 0.25% Triton buffer in 1X PBS (Fisher Scientific # PR-H5142) for 5 min at room temperature. Cells were then washed three times with 1X PBS prior to blocking with 3% bovine serum albumin (BSA, Sigma # A7906, Saint Louis, MO) in 1X PBS. Cells were then stained with a monoclonal mouse antibody against the EBOV matrix protein, VP40 (VP40 B-MD04-BD07-AE11; generous gift of the United States Army Medical Research Institute of Infectious Diseases, Fort Detrick, Frederick, MD, USA) (commercially available from IBT Bioservices, catalog # 0201–018) diluted to 0.002 mg/mL in 3% BSA; washed, and stained with an Alexa Fluor 488 fluorescently labeled goat anti-mouse IgG (H+L) highly cross-adsorbed secondary antibody (Thermo Fisher Scientific #A11029) and nuclear stain Hoechst 33342 (Thermo Fisher Scientific #H3570). Signals for each sample/replicate were measured using the Operetta CLS High-Content Analysis System (Perkin Elmer, Waltham, MA).

### Chemiluminescent enzyme-linked immunosorbent assay

As an alternative to Operetta signal detection, a previously described chemiluminescent enzyme-linked immunosorbent assay based on anti-EBOV VP40 antibody VP40 B-MD04-BD07-AE11 staining followed by staining with a horseradish peroxidase (HRP)-conjugated goat anti-mouse secondary antibody (SeraCare, Milford MA, #074–1802) was used for detection of EBOV [13].

### Human clinical samples

Human serum samples from the Partnership for Research on Ebola Virus in Liberia III (PREVAIL III, ClinicalTrials.gov identifier NCT02431923) were used to assess the assay. PREVAIL III is a natural history study of Liberian survivors of the 2013–2016 EVD epidemic in Western Africa and their close contacts (defined to be household members, friends or neighbors who were in close proximity to EVD survivors at the time of or since the EVD epidemic and sexual partners of survivors since the EVD epidemic). Samples from survivors were obtained about a year after acute infection (median 358 days with quartiles 313 and 405 days). The FANG ELISA has been used to determine the serostatus and anti-EBOV antibody titer in samples obtained during PREVAIL III study. [9, 14, 15]. Of those who enrolled as survivors, 13% were seronegative, and of those who enrolled as close contacts, 11% were seropositive. For this study, 20 samples from the PREVAIL III study were obtained: 11 from participants who enrolled as survivors (3 of these samples were seronegative by the FANG assay) and 9 from participants who enrolled as contacts (5 of these samples were seropositive by the FANG assay). All adults provided written consent and all children were consented by their parents and/or provided consent as appropriate. The study was approved by the National Institute of Allergy and Infectious Diseases (NIAID) Institutional Review Board and National Research Ethics Board of Liberia.

To perform FRNA, human clinical samples were heat-inactivated at 56°C for 1 h prior to use, and then serially diluted (1:2) over 10 dilutions in serum-free DMEM. Samples were then treated like surrogate samples as described above and staff were blinded to the results from the FANG ELISA and all other information about the samples.

### Calculations and statistics

Two aspects were used for interpretation of the FRNA: determination of antibody positivity and quantification of the level of antibody positivity. Analysis began by comparing the number of cells staining positive using the lowest dilution of a sample to the number of cells staining positive in EBOV-only wells. This comparison was made by statistically testing the null hypothesis that the proportion of EBOV-infected cells in a well containing the lowest dilution of a sample is equal to some constant *c* times the mean proportion of EBOV-infected cells in EBOV-only wells against the alternative that there are more EBOV-infected cells in EBOV-only wells (see the Supporting information for a description of how to estimate *c*). If this test is conducted with a significance level of α, then the specificity of the assay is given by 1 - α. We set α = 0.01 to fix the specificity of the assay at 99%.

To quantify the level of antibody positivity, the half maximal inhibition of relative fluorescence intensity (FRNA_50_) is defined as the largest dilution so that the proportion of infected cells is less than or equal to 50% of the proportion of infected cells in the virus-only wells. If the lowest dilution has cells that are more than 50% positive, then the assay is left-censored, and we define the titer to be half of the lowest dilution. If the proportion of infected cells at the highest dilution is less than half of the proportion of infected cells in the virus-only wells, then the titer is right-censored, and we define the titer to be given by this highest dilution. A simple probability model based on the binomial distribution, described in Supporting information, was used to derive expressions for the sensitivity and specificity of this procedure and the coefficient of variation of the FRNA_50_ in Section 3.

Linear regression models were used to determine the impact of fields of cells on the boundaries of the wells, the total number of cells, the number of EBOV-positive cells, and the percentage of EBOV-positive cells. Parameter estimates in these models were obtained using generalized estimating equations. The latter three variables were logarithmically transformed and served as the response variable in linear models, which included the number of plates (4 plates), replicates (3 replicates), dilution, the interaction between dilution and sample and an indicator of a boundary field. To test for the impact of low cell counts in wells, FRNA_50_ values were computed with and without low cell count fields for all possible combinations of field use. To determine the impact of varying the number of fields, various numbers of fields were used and the mean coefficient of variation for each number of fields was computed. All statistical calculations were conducted using R and SAS.

## Results

### Impact of cell numbers per field on FRNA-derived antibody titers

**Fig 1:**
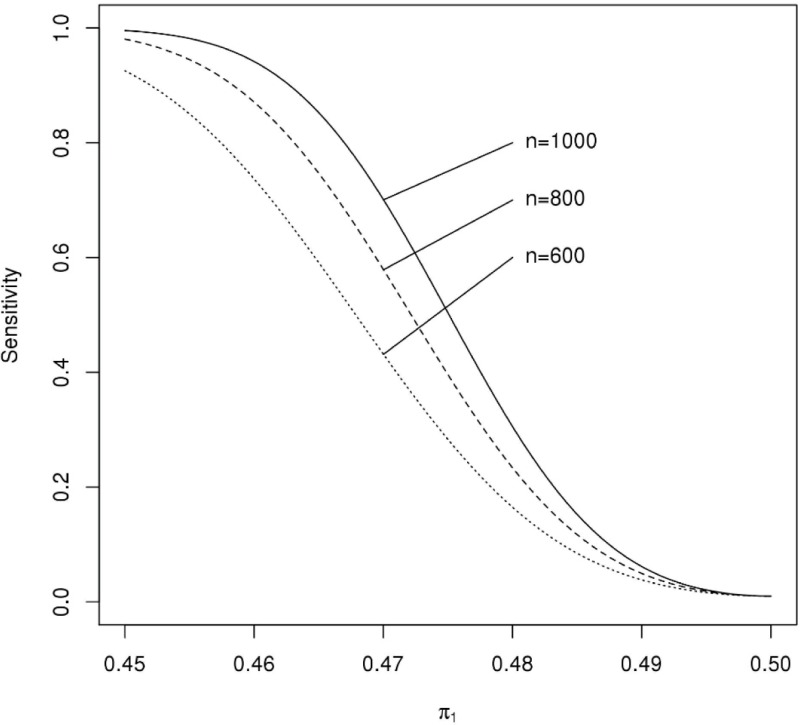
Assay sensitivity. The sensitivity of the assay as it depends on the number of cells per filed (n) and the probability that a cell is infected at the lowest dilution (π_1_) given that the probability a cell is infected in the virus only wells is 50% and c = 1.

As the number of cells per field can be influenced by a variety of factors, we used the probability model described in the Supporting information to examine how the sensitivity of the assay depends on the number of cells per field. We have found that the best quantity of virus to use to ensure high quality imaging is the amount that is needed to infect about 50% of the cells in the virus-only well, so the results presented here make that assumption. The FRNA sensitivity depends on the number of cells per field. For example, using the 3 values of 600, 800 and 1,000 for the number of cells per measured field, Fig 1 shows how the sensitivity depends on the probability that a cell is EBOV-infected in the lowest dilution well. Consequently, a substantial loss of sensitivity can occur if as few cells as 600 cells are present in a field if the probability that a cell is EBOV-infected at the lowest dilution well is close to the value in EBOV-only wells (i.e., representing a weak antibody response in the patient from whom a sample was taken). If the probability of infection in the lowest dilution well is 0.46 with only 600 cells, the sensitivity would be about 80%, whereas with 1,000 cells, the sensitivity is over 95% (Fig 1).

We used a similar approach to examine how the coefficient of variation depends on the number of cells per field. In comparing 1,000 to 800 cells, the mean ratio of the coefficient of variation is 0.74. Similarly, in comparing 800 to 600 cells, the mean of this ratio is 0.72. Thus, the coefficient of variation is about 75% of that which is obtained with 200 fewer cells. Since the power of statistical tests depend on the coefficient of variation, lower numbers of cells could reduce the power of an investigation which used the quantitative estimate provided by the assay as the primary endpoint (for example, in vaccine trials).

**Fig 2:**
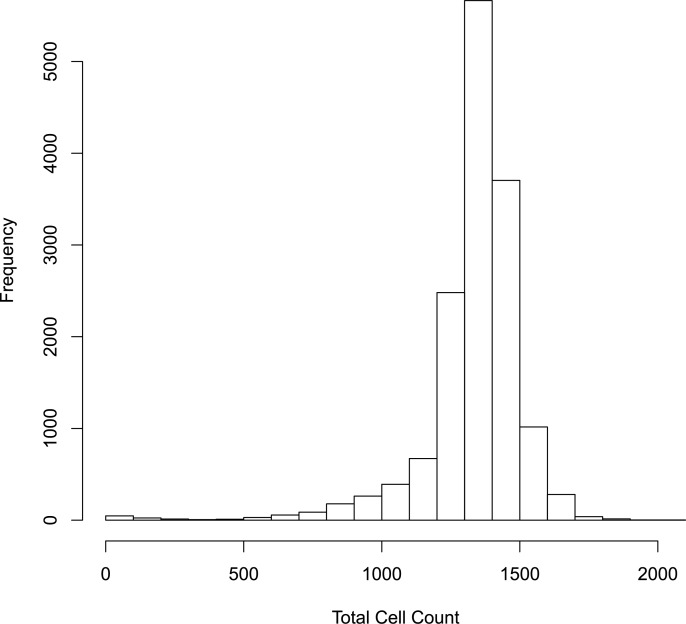
Total cell count distribution. Distribution of total cell counts across all samples, plate columns, fields, and replicates.

**Fig 3:**
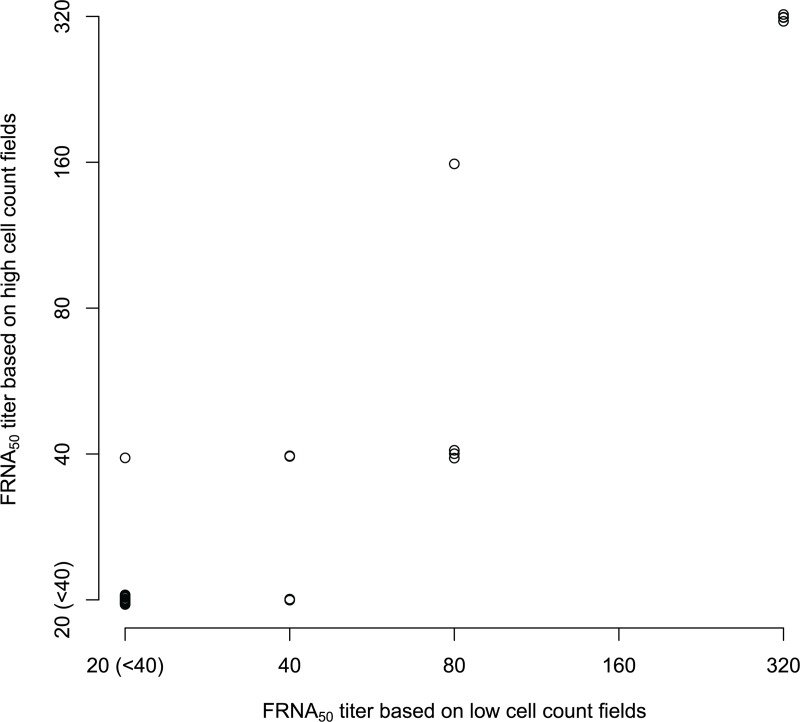
FRNA_50_ titer estimate in fields with varying cell count. Comparison of FRNA_50_ titer estimates calculated using low cell count and high cell count fields. For each plate and column replicate, the field corresponding to either the minimum or maximum total cell count was used to estimate functional titers.

Using a unique combination of row, plate, replicate, dilution, and field set to analyze 14975 observations, 708 (4.73%) observations were based on a total cell count of 1,000 or lower per field. Therefore, few fields were potentially impacted by low cell counts using the clinical samples. Fig 2 shows a histogram of the number of cells per well for the data presented here. Moreover, fields with low cell counts had little impact on the estimates of antibody titers, as 90% of the samples had similar FRNA_50_ values (Fig 3 compares these results). These results suggest that most of these samples that are positive have effective antibody response compared to the virus-only wells. Whereas the model indicates that low cell counts are potentially a problem, in practice the extent of this problem for the samples studied here is minimal. If experimental conditions are such that many fields have low cell counts, potential for a lack of sensitivity and a high coefficient of variation for titers exists. For these reasons, we recommend seeding wells so that there at least 1,000 cells per field.

## Impact of number of fields per well on FRNA-derived antibody titers

With a high-content imaging system, one can assess multiple fields-of-interest in a well to determine the proportion of cells that are EBOV-infected. Assessing multiple fields increases the precision of cell number estimates but takes more time to analyze.

**Fig 4:**
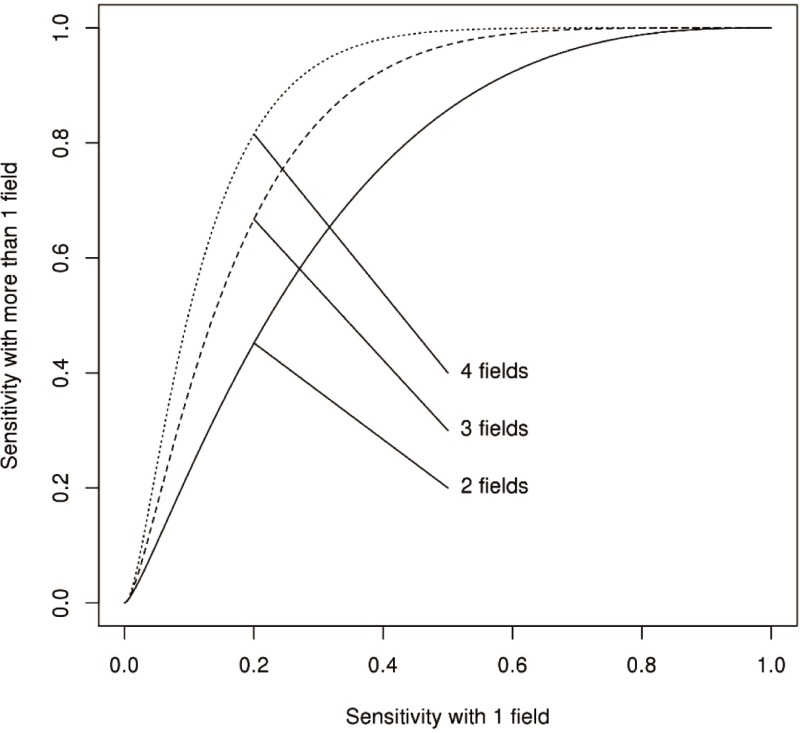
Assay sensitivity comparisons. The improvement in sensitivity that is possible by using multiple fields.

The probability model described previously can also be used to examine the impact of the number of fields on the sensitivity of the assay and the coefficient of variation of the estimated FRNA_50_ values. Sensitivity of the assay improves by using more than 1 field in determining the FRNA_50_ values (Fig 4). If experimental conditions and a sample’s functional antibody response are such that high sensitivity is obtained with 1 field, little benefit is gained from the use of additional fields. However, the use of additional fields is a relatively easy method to increase sensitivity when sensitivity might otherwise be lacking. For example, if the sensitivity of the assay with 1 field is only 50%, the sensitivity of the assay increases to over 80% by just using 1 more field and to over 90% by using 3 fields. The sensitivity is quite high for detecting even a difference of 5% in the probability of cell infection at the lowest dilution compared to the virus-only well (Fig 1). Hence, unless there is less than a 3% difference in these probabilities, a single field with 1,000 cells will provide adequate sensitivity. A single field will not have adequate sensitivity if there is only a 3% difference in these two probabilities; in this case, the using 4 fields will result in an assay with over 99% sensitivity.

**Fig 5:**
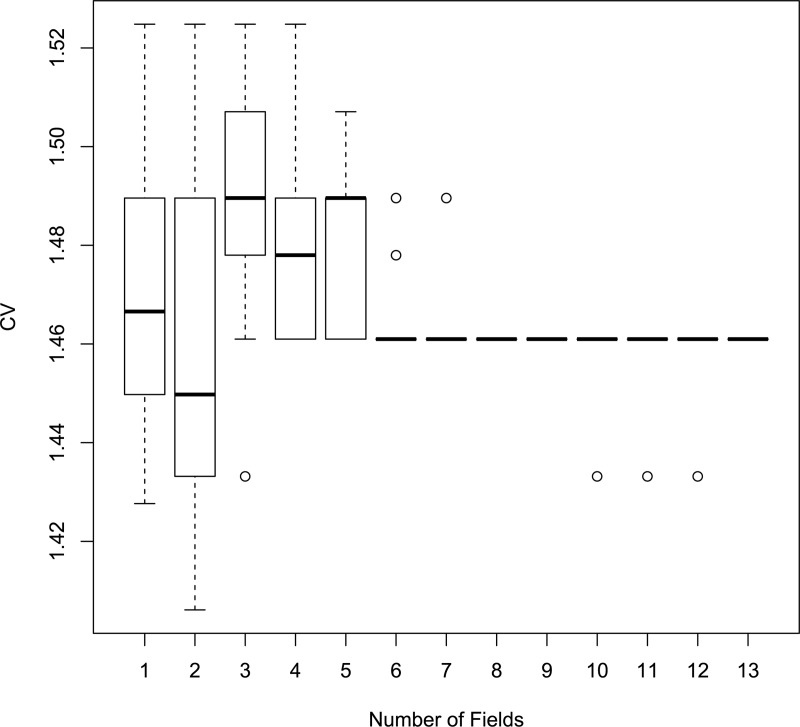
Coefficient of variation. Distribution of the coefficient of variation by the number of fields used to calculate FRNA_50_ titers.

We also used the samples from the PREVAIL III study to investigate how the estimated infection probabilities influence the coefficient of variation. A single field was selected at random and the proportion of infected cells at all dilutions for all samples was computed, including the virus-only wells. We then used these proportions to compute the coefficient of variation using the approach provided in the Supporting information. The effect of increasing the number of fields is to increase the total number of cells by an integer. The coefficient of variation for each sample decreases as the number of fields increases (Fig 5). The viral titer for most samples is highly accurate with even a single field, but some samples have quite variable titers. These samples have probabilities of cell infection that are nearly flat over several dilutions, resulting in highly variable titers. To ensure only that only a small fraction of samples have highly variable titers, we recommend the use of 4 fields.

## Impact of edge effects on FRNA-derived antibody titers

**Fig 6:**
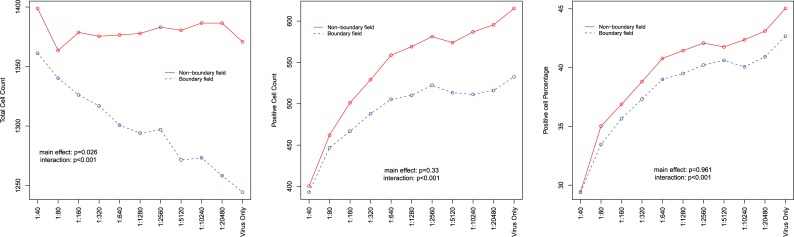
Comparison of cell count by field location. Comparison of total cell count, positive cell count and positive cell percentage between boundary fields and non-boundary fields by plate column. Cell counts and percentages are averaged across samples, replicates and fields. Statistical significance was assessed via GEE linear regression models that controlled for the effects of plate and replicate and adjusted for repeated measures among samples.

When fields are on the boundary of a well, fluctuations in cell numbers occur. Statistically significant differences attributable to boundary effects were detected across a wide range of conditions. Moreover, complex interactions between dilutions and boundary fields were detected across plates and replicates (Table 1 and Fig 6). A 6% mean reduction in the number of cells in a boundary field was found with a range from 3–9%. Unexpectedly, boundary fields (curved edge in a cell culture well) were identified in areas considered to be full interior fields. We assume slight variations in plate placement in the imager may result in shifting boundary fields. These results indicate that data should not be collected from boundary fields when conducting this assay.

**Table 1:** Comparison of total cell count, positive cell count and positive cell percentage between boundary fields and non-boundary fields by plate column.

Plate column	Total Cell Count by field type		Positive cell count by field type		Positive cell percentage by field type	
	Non-boundary	Boundary	Non-boundary	Boundary	Non-boundary	Boundary
1:40	1398.8 (197.5)^a^	1361.2 (196)	400.3 (273.2)	393.1 (271.7)	29.4 (20.1)	29.4 (20.1)
1:80	1363.4 (212.5)	1340.3 (139.9)	462 (259.4)	446.8 (248.3)	35 (19.6)	33.5 (18.4)
1:160	1378.6 (132.2)	1326.1 (150.5)	501.2 (229.9)	466.7 (226)	36.9 (17.1)	35.7 (17.1)
1:320	1375.4 (127.3)	1316.9 (144.9)	529.1 (196.2)	488 (187.3)	38.8 (14.3)	37.3 (13.9)
1:640	1376.5 (104.2)	1300.8 (156.8)	558.7 (133.9)	505.4 (144.4)	40.8 (9.9)	39 (10.2)
1:1280	1377.8 (93.9)	1294.1 (161.7)	569 (123.7)	510.2 (136.5)	41.4 (9.1)	39.5 (9.2)
1:2560	1383 (110)	1297 (158.2)	581.2 (123.2)	522.3 (136.4)	42.1 (8.5)	40.2 (8.9)
1:5120	1380.4 (114.8)	1271.5 (202.3)	574 (116.7)	513.3 (142.5)	41.8 (8.5)	40.6 (9.5)
1:10240	1386.5 (92.4)	1273.4 (186.5)	586.9 (118.9)	511.3 (137.6)	42.4 (8.4)	40.1 (8.6)
1:20480	1386.5 (112.3)	1258.4 (220)	595.4 (115.2)	516.1 (149.8)	43.1 (8.2)	40.9 (9)
Virus Only	1370.7 (119)	1244.5 (240.7)	615.3 (134)	532.6 (166.6)	45 (9.4)	42.7 (9.8)
Boundary field effect *p*-value^b^	0.026		0.33		0.961	
Boundary field×column interaction *p*-value^b^	*<*0.001		*<*0.001		*<*0.001	

^a^Means and standard deviations are provided. Cell counts and percentages are averaged across samples, replicates and fields.

^b^Statistical significance was assessed via GEE linear regression models that controlled for the effects of plate and replicate and adjusted for repeated measures among samples.

## FRNA-determined EBOV-neutralizing antibody titers correlate with the clinical disease stage of humans

Using the parameters determined above using surrogate samples, we applied the FRNA to a 20 blood samples obtained from humans, 11 of whom had been diagnosed with EVD by the Liberian Ministry of Health at the time of their acute infection, and 13 of whom displayed evidence of antibodies using the FANG ELISA. To test for presence of anti-EBOV antibodies in these samples, we need to first estimate *c* (see the Supporting information for details) and then conduct the resulting test. FRNA detected EBOV-neutralizing antibodies in 10 of the 13 FANG ELISA-positive samples and in 1 of the 7 FANG ELISA-negative samples (Table 2). If the FANG-ELISA is the current standard for antibody testing, then the FRNA has a sensitivity of 76.9% (95% confidence interval 46.2%-95.0%) and a specificity of 85.7% (95% confidence interval 42.1%-99.6%). There was strong agreement of the FRNA titer and the FANG ELISA titer as indicated by a Spearman correlation of 0.95 that was statistically significant (*p* <0.001). Interestingly, two of five (40%) seropositive close contacts of an EVD survivor appear to have a functional antibody response, whereas 1 of 3 (33%) of the seronegative survivors tested appears to have such a response.

**Table 2:** Comparison of FANG assay results and FRNA_50_ titers computed by the Operetta and Chemi methods.

	Operetta FRNA_50_^a^	Chemi FRNA_50_^a^	FANG	EVD Ab status per FRNA assay
Seropositive Survivors				
1-A	80	80	57688.49	Ab positive
1-B	40	40	35621.13	Ab positive
1-C	20	20	13369.94	Ab positive
1-E	80	80	39196.41	Ab positive
2-A	40	80	47295.02	Ab positive
2-B	20	40	59955.6	Ab positive
2-D	160	320	71631.49	Ab positive
2-E	40	40	19714.9	Ab positive
Seronegative Close Contacts				
4-B	20	10	9.48	Ab negative
4-C	20	10	10.64	Ab negative
4-D	20	10	11.63	Ab negative
4-E	20	10	14.88	Ab negative
Seropositive Close Contacts				
3-A	20	40	14731.89	Ab positive
3-B	20	10	851.91	Ab negative
3-D	40	80	21381.32	Ab positive
3-E	20	10	44945.63	Ab negative
4-A	20	10	713.32	Ab negative
Seronegative Survivors				
1-D	20	10	358.08	Ab positive
2-C	20	10	130.65	Ab negative
3-C	20	10	58.44	Ab negative
Control Samples (Plate 1 Only)				
VLP ab IBT	320	640	NA	NA
SAB-301 anti-MERS antibody	20	10	NA	NA
Human Negative CTRL #7	20	10	NA	NA
Spearman Correlation—Chemi				
*ρ* *p*-value	0.92 *<* 0.0001	– –	0.809 *<* 0.0001	NA
Spearman Correlation—FANG				
*ρ* *p-*value	0.675 0.0011	0.809 *<* 0.0001	– –	NA

^a^The Operetta FRNA_50_ titers and EVD Ab status results were calculated using four non-boundary fields. Operetta titers of 20 and Chemi titers of 10 indicate an estimate below 40 and 20, the LODs of the Operetta and Chemi methods, respectively.

## Discussion

Here we report an alternative, simplified, semi-automated fluorescence-reduction neutralization assay (FRNA) based on the classical PRNT. In this assay, live EBOV is incubated in the presence of serial dilutions of clinical test sample prior to plating on EBOV-permissive Vero E6 cells in 96-well plates. EBOV not neutralized by the antibodies in the clinical test sample will then infect cell monolayers. After 48 h of incubation, the cells are fixed and stained for expressed EBOV antigen. The assay readout is adaptable for measuring the number of EBOV-infected cells using a high-content imaging system or for measuring total luminescence using a plate reader. The antibody neutralization titer can be calculated by comparing the number of EBOV-infected cells in each sample dilution well to the EBOV-only control wells (no test sample) or percent change in fluorescence compared to EBOV-only (no sample) wells.

Establishment of this assay is associated with some caveats. For instance, we previously reported that the EBOV-permissiveness of Vero E6 cells decreases over increasing cell passages [13]. Consequently, FRNA establishment needs to be preceded by assessment of each cell line and passage number for permissiveness to individual EBOV stock infection. Furthermore, FRNA settings should be chosen that include an appropriate number of randomized fields containing an appropriate number of cells and exclude edge fields. We recommend the use of at least 4 fields containing >1,000 cells each which results in over 95% sensitivity with a specificity of at least 99%. Finally, we recognize the FANG ELISA and the FRNA measure two different fractions of antibodies in a samples—the FANG ELISA measures total anti-EBOV glycoprotein (GP1,2) IgG antibodies whereas the FRNA measures total neutralizing antibodies. These two assays provide complementary, and not competing, data.

We then compared the EBOV-neutralizing antibody titers in samples of EVD survivors to antibody titers obtained for the same samples using the FANG ELISA. In contrast to FRNA, which only measures EBOV-neutralizing antibodies, FANG ELISA determines total anti-EBOV glycoprotein (GP_1,2_) IgG antibodies. Thus, a sample with neutralizing antibodies should also be FANG ELISA-positive, but not necessarily vice versa. Indeed, in five FANG ELISA-seropositive close contacts of EVD patients, the FRNA only identified two samples as positive. On the other hand, a FANG ELISA-seronegative survivor had a low titer of neutralizing antibodies in the FRNA assay. Because the FANG ELISA does not utilize infectious virus in the assay, the FANG ELISA can easily screen samples for total IgG antibodies in a sample as long at the antibodies react with antigen utilized in the ELISA. However, the FRNA, which requires testing in biosafety level 4 (BSL 4) containment, provides insight into the functionality of the complete, not only IgG, antibody response in an individual sample. Alternatively, if convalescent serum is mismatched to the virus used in the FRNA, cross neutralization can be measured.

The FRNA assay described here can easily adapted for serological studies among humans and wild or domestic animals to better characterize EVD epidemiology, survivor humoral responses, antibody-based therapeutic efficacy, or vaccine immunogenicity. Unlike standard ELISA assays, which only detect one type of antibody (IgG for example), the FRNA assay detects all neutralizing antibodies throughout antibody maturation. The assay can be semiautomated allowing for high throughput in the BSL4 setting for screening large numbers of antibody therapeutics or serum samples from clinical trials.

## Supporting information

S1 File Supporting materials. PONE-D-19-11230R1_FTC2_Supporting_Materials.
(DOCX)
