# Supplementary material for: Scalable, semi-automated fluorescence reduction neutralization assay for qualitative assessment of Ebola virus-neutralizing antibodies in human clinical samples
Source: PLoS One. 2019 Aug 27;14(8):e0221407. doi: 10.1371/journal.pone.0221407 (PMC6711594; doi:10.1371/journal.pone.0221407)
Supplement: S1 File — PONE-D-19-11230R1_FTC2_Supporting_Materials. (DOCX) [file pone.0221407.s001.docx]

**Supporting Materials**

Here we provide details about the probability model we use to investigate the sensitivity, specificity and properties of the estimated FRNA_50_. We let *x_i_* represent the number of infected cells out of a total of *n_i_* cells in a sample at dilution *i*, *p_i_* represent the corresponding

sample proportions and *π_i_* the corresponding probabilities (*x_1_* is the result at the lowest

dilution). We let *v_0_* represent the mean proportion of infected cells across the 8 virus only wells,

and we assume the probability that a cell is infected in the virus only wells is *π_0_*. Here we make the simplifying assumption that all fields have the same number of cells *n* and that all

experiments are done in triplicate so that the sampling distribution of *p_i_* is approximately normal

with mean *π_i_* and variance *π_i_* (1- *π_i_* )/(3*n*) and the sampling distribution of *v_0_* is also approximately normal with mean *π_0_* and variance *π*_0_ (1- *π*_0_ )/(24*n*).

**Specificity**

To make a call for the presence or absence of antibody we propose testing the null hypothesis H_0: *π*_1_=*c* *π*_0_ for some *c*. Since the sample proportions are approximately normal we can use the following test statistic to test this null hypothesis:

T=(*c v*_0_*-p*_1_)/(*c*^2^ *v*_0_(1-*v*_0_)/(24*n*)+*p*_1_(1-*p*_1_)/(3*n*))^0.5^.

If *Z* is a standard normal random variable then we define *P(Z < z_x_)=x*. Thus if *T>z*_1-α_ then we conclude that antibodies are present and the sample is seropositive. This procedure will ensure that the specificity is at least 100(1-α)%.

**Sensitivity**

The sensitivity of the test corresponds to the power of the statistical test, hence using the usual

approach to computing the power and letting

*s_d_*=(c^2^ *π*_0_ (1- *π*_0_)/(24*n*)+ *π*_1_(1- *π*_1_)/(3*n*))^0.5^,

and Φ(x) the standard normal cumulative distribution function (cdf), we find that the sensitivity is given approximately by 1-Φ(*z*_1-α_-(c *π*_0_- *π*_1_)/*s_d_*). This assumes that a single field was used, If additional fields are used that will increase *n* by some integer, if *n_f_* fields are used and if we let s=(c^2^ *π*_0_ (1- *π*_0_)/24+ *π*_1_(1- *π*_1_)/3)^0.5^ we find that the sensitivity is 1-Φ(*z*_1-α_-(*n_f_n*)^0.5^(c *π*_0_- *π*_1_)/*s*).

**Coefficient of variation**

If the sample is seropositive we would like to estimate the antibody level in the sample using the

FRNA_50_. Given the algorithm stated in the text, FRNA_50_ values are discrete random variables that take 1 of 11 possible values, hence computing the moments of this variable is relatively straightforward.

**Estimation of *c***

To determine a value for the parameter *c* that enters the statistical test used for determining if a sample is EBOV-positive, we regress the proportion of EBOV-infected cells on the logarithm (base 2) of the dilution for each sample. Then we estimate *c* by computing the mean of the ratio of the of proportion of EBOV-infected cells in the lowest dilution sample well to the

proportion of EBOV-infected cells in EBOV-only wells for those samples with estimated regression coefficients within 2 standard errors of 0. This will provide a consistent estimate of *c*.
